# Locked-in syndrome responding to endovascular treatment

**DOI:** 10.1136/jnis-2022-019112

**Published:** 2022-08-19

**Authors:** Jiaxing Song, Jiacheng Huang, Linyu Li, Jie Yang, Chengsong Yue, Shuai Liu, Weilin Kong, Xiaojun Luo, Jiasheng Liao, Jie Du, Bo Song, Jiazuo Liu, Xiaolong Tian, Xiaolin Tan, Fengli Li, Wenjie Zi

**Affiliations:** 1 Army Medical University Xinqiao Hospital Department of Neurology, Chongqing, China; 2 Department of Cerebrovascular Diseases, Guangyuan Central Hospital, Guangyuan, Sichuan, China; 3 Department of Neurology, Suining No.1 People's Hospital, Suining, China; 4 Department of Neurology, The People's Hospital of Kaizhou District, Chongqing, Chongqing, China; 5 Department of Neurology, Meishan People's Hospital, Meishan, Sichuan, China; 6 Department of Neurology, Dazhou Central Hospital, Dazhou, Sichuan, China; 7 Department of Neurology, Qianjiang Central Hospital, Chongqing, Chongqing, China; 8 Department of Neurology, Meishan Second People's Hospital, Meishan, Sichuan, China

**Keywords:** Stroke, Intervention, Thrombectomy

## Abstract

**Background:**

Locked-in syndrome (LiS) is a rare and devastating condition in patients with acute basilar artery occlusion. However, the benefits of endovascular treatment (EVT) for LiS remain unclear.

**Objective:**

To assess the outcomes associated with EVT and identify the factors associated with outcomes of LiS.

**Methods:**

We used the data of the Endovascular Treatment for Acute Basilar Artery Occlusion Study Registry (BASILAR) from 47 tertiary stroke centers in China. The included patients had LiS and received EVT or standard medical treatment (SMT) alone. The primary outcome was improvement in the modified Rankin Scale (mRS) score at 90 days.

**Results:**

Among the 120 patients with LiS, 92 (76.7%) received EVT and 28 (23.3%) received SMT. Compared with SMT, EVT was associated with improved mRS score (common OR (cOR)=2.68 (95% CI 1.16 to 6.20); p=0.02) and decreased mortality (aOR=0.35 (95% CI 0.13 to 0.90); p=0.03). Moreover, the benefit of EVT for LiS was sustained for at least 1 year (p=0.008). Higher baseline posterior circulation Alberta Stroke Prognosis Early CT Score (pc-ASPECTS, aOR=2.04 (95% CI 1.34 to 3.10); p<0.001) and absence of pneumonia (aOR=0.26 (95% CI 0.08 to 0.90); p=0.03) were significantly associated with favorable functional outcome at 90 days in patients who received EVT, while lower pc-ASPECTS (aOR=0.52 (95% CI 0.36 to 0.76); p<0.001) was associated with increased 90-day mortality.

**Conclusions:**

This study found that EVT was associated with favorable functional outcomes and decreased mortality among patients with LiS. Baseline pc-ASPECTS and pneumonia were independent predictors of outcomes.

**WHAT IS ALREADY KNOWN ON THIS TOPIC**

- Locked-in syndrome (LiS) is a rare and devastating condition in patients with acute basilar artery occlusion (ABAO). However, the benefits of endovascular treatment (EVT) for LiS remain unclear.

**WHAT THIS STUDY ADDS**

- This study aimed to assess the outcomes associated with EVT and identify the factors associated with outcomes of LiS. This study found that EVT was associated with favorable functional outcomes and decreased mortality among patients with LiS. Baseline posterior circulation Alberta Stroke Prognosis Early CT Score and pneumonia were independent predictors of outcomes.

**HOW THIS STUDY MIGHT AFFECT RESEARCH, PRACTICE OR POLICY**

- Our study provides evidence supporting the safety and efficacy of EVT for patients with LiS secondary to ABAO who can be treated within 24 hours of the estimated occlusion time.

## Introduction

Locked-in syndrome (LiS) is a rare and devastating condition that results in tetraplegia, lower cranial nerve paralysis, and anarthria, with preserved cognition, vertical gaze, and upper eyelid movements.^1^ There are three subtypes of LiS: classic, incomplete, and total. The classic form presents with quadriplegia, anarthria, preserved consciousness, and vertical eye movements.^2^ In incomplete LiS, the patient has residual movements apart from the eyelid and vertical eye movements. Total LiS is characterized by complete immobility and inability to communicate. LiS is most commonly caused by an infarct of the ventral pontine due to occlusion or thrombosis of the basilar artery, which interrupts the corticospinal and corticobulbar fibers to the lower cranial nerves, but spares the reticular activating system. Other causes have been cited in the literature, such as tumors or encephalitis of the brainstem, traumatic lesions, central pontine myelinolysis, multiple sclerosis, and drug intoxication.^3 4^

Although the clinical features, diagnosis, and classification of LiS have been established, its treatment and prognosis remain largely unknown. Several case studies have reported the prognosis of this illness, the largest of which showed a severely disabled state or death.^5^ However, there have been several reports of patients with substantial clinical improvement, which suggests that early stroke treatment, such as endovascular mechanical thrombectomy, intravenous thrombolysis, and intra-arterial thrombolysis with tissue plasminogen activator, may enhance the possibility of a greater recovery for patients with LiS secondary to acute basilar artery occlusion (ABAO).^6 7^

To our knowledge, information on rapid endovascular treatment (EVT) for patients with LiS is also scarce, especially regarding the short- and long-term prognoses for these patients. This study aimed to provide additional information about LiS secondary to acute basilar artery occlusion. In this study, we sought to explore the short- and long-term outcomes of EVT for patients with LiS secondary to ABAO.

## Methods

### Study design, setting, and data collection

This study used the registry dataset of the Endovascular Treatment for Acute Basilar Artery Occlusion Study Registry (BASILAR), which was a multicenter, observational study including 829 consecutive patients confirmed to have ABAO between January 2014 and May 2019 in 47 tertiary stroke centers across 15 provinces in China. The details of the study protocol have been published previously.^8^ The institutional review boards of each center approved the study protocol. Written informed consent was obtained from all patients or their legally authorized representatives, according to the Declaration of Helsinki.

### Participants

We included consecutive study participants from BASILAR if they fulfilled the following criteria: (1) age ≥18 years; (2) presentation with acute, symptomatic, angiographically confirmed BAO within 24 hours of the estimated occlusion time or, if not known, at the last time the patient was observed to have no symptoms before the onset of stroke symptoms; and (3) underwent intravenous thrombolysis within the thrombolysis time window.

The following are the characteristics of LiS. In patients with classic LiS, the main clinical manifestations are: (1) complete quadriplegia (grade 0 muscle strength in the limbs); (2) complete anarthria and were unable to speak; (3) preserved vertical movement of the eye and blinking only (when the doctors obtain the National Institutes of Health Stroke Scale (NIHSS) score or other evaluation); (4) patients have preserved consciousness. In patients with incomplete LiS, the main clinical manifestations are: (1) tetraparesis (part of the muscle strength of the limbs is preserved); (2) partial anarthria and hypophonia; (3) residual movements besides eyelid, vertical eye movements, and blinking (when the doctors obtain the NIHSS score or other evaluation); (4) preserved consciousness.

The participants were divided into an EVT group (ie, those receiving standard medical treatment (SMT) in addition to mechanical thrombectomy with stent retrievers and/or thromboaspiration, balloon angioplasty, stenting, intra-arterial thrombolysis, or various combinations of these approaches) and an SMT group (ie, those receiving SMT alone, including antiplatelet and anticoagulation treatment, intravenous thrombolysis, or a combination of these therapies). The exact therapeutic strategies used were at the discretion of the local neurointerventionist.

### Data collection

We recorded the patients’ baseline characteristics, stroke risk factors, laboratory findings, estimated time of BAO, neurological deficits at the time of treatment, pretreatment and post-treatment imaging findings, type of treatment, EVT characteristics, complications, presumed cause of stroke, and functional outcomes at 90 days and 1 year.

The presumed cause of stroke was assessed based on the Trial of Org10172 in Acute Stroke Treatment (TOAST) classification.^9^ The NIHSS was used to assess neurological deficits at the time of treatment. The posterior circulation Alberta Stroke Prognosis Early CT Score (pc-ASPECTS; range 0–10, with scores ≥8 indicating favorable outcomes) was used to quantify the ischemic changes on baseline imaging.^10^ The posterior circulation collateral score represented the collateral circulation status based on the presence of potential collateral pathways on CT angiography,^11^ and the Basilar Artery Treatment and Management (BATMAN) score was used to assess the status of the posterior circulation collaterals.^12^ The estimated time of BAO was defined as the time of symptom onset, as described by the patient or witness, consistent with the clinical diagnosis of BAO, based on the judgment of the treating physician, or, if the exact time was not known, recorded as the last time the patient was seen well.

### Outcome measures

The primary outcome of clinical efficacy was the modified Rankin Scale (mRS) score at 90 days, as assessed by trained local neurologists who were blinded to the treatment group assignments. The mRS is an ordinal scale that ranges from 0 (no symptoms) to 6 (death). Secondary outcomes included the mRS score at 1 year, category scores of the mRS at 90 days and 1 year (0 or 1 (excellent outcome), 0–2 (good outcome, indicating functional independence), and 0–3 (favorable outcome)), mortality at 90 days and 1 year, and symptomatic intracranial hemorrhage based on the Heidelberg bleeding classification. Successful recanalization was defined as a modified Thrombolysis in Cerebral Infarction (mTICI) score of 2b–3.^13^ This scale ranges from 0 (no reperfusion) to 3 (complete recanalization), based on angiographic data.

### Radiologic assessment

Correlative neuroimaging data were analysed by a neuroimaging core laboratory whose members were independent, experienced neuroradiologists who were unaware of the treatment group assignments, clinical data, and outcomes. Each imaging scan was separately interpreted by two trained neuroradiologists. For cases with disagreement, decisions were made by a third experienced neuroradiologist. The detailed methods of the BASILAR have been reported previously.

### Statistical analysis

Categorical and binary variables were compared using the χ^2^ or Fisher exact tests, while continuous variables were compared using Student’s t-test for variables with normal distributions and the Mann-Whitney U test for variables without normal distributions. For baseline characteristics and outcomes, data are presented as medians (interquartile ranges (IQRs)) or numbers with percentages, unless otherwise indicated. We compared binary outcomes (ie, favorable outcome vs poor outcome) for the EVT versus SMT groups and performed shift analysis on the mRS score using multivariable binary logistic regression and ordinal logistic regression analysis. For regression analyses with favorable outcomes and mortality, we adjusted for the following prognostic factors: age, diabetes mellitus, and baseline NIHSS score. Logistic regression analysis was performed to identify independent factors of outcomes in patients with LiS who underwent EVT. All-cause mortality was assessed using the Kaplan-Meier method.

Statistical analyses were performed using SPSS version 26 (IBM, Armonk, New York, USA). The level of statistical significance was set at a two-tailed p value of <0.05. We excluded patients with missing essential data from our analysis; thus, we did not impute missing data.

## Results

### Baseline characteristics

A total of 829 patients with acute BAO from 47 stroke sites in China were enrolled in BASILAR, of whom 120 had LiS (online supplemental figure I). Among patients with LiS, the median (IQR) age and baseline NIHSS score were 67 (57–75) years and 23 (18–27), respectively. The included patients comprised 29 (24.2%) women and 91 (75.8%) men; 92 patients (76.7%) received EVT, and 28 patients (23.3%) received SMT. A comparison of baseline characteristics between patients with EVT and SMT is provided in table 1.

10.1136/jnis-2022-019112.supp1 Supplementary data

**Table 1:** Baseline characteristics of patients with locked-in syndrome

Variables	All patients	SMT	EVT	P value
	120	28	92	
Age, years, median (IQR)	67.00 (57.0–75.0)	69.00 (60.0–75.0)	65.00 (57.0–75.0)	0.529
Sex, male, n (%)	91 (75.8)	20 (71.4)	71 (77.2)	0.712
SBP, mm Hg, (mean (SD)	156 (25.0)	162 (27.0)	154 (24.0)	0.117
Smoking (%)	39 (32.5)	7 (25.0)	32 (34.8)	0.461
NIHSS score at baseline, median (IQR)	23.0 (18.0–27.0)	23.0 (18.0–26.0)	24.0 (19.0–27.0)	0.443
pc-ASPECTS at baseline, median (IQR)	8.0 (7.0–9.0)	8.0 (6.0–8.0)	8.0 (7.0–9.0)	0.131
pc-CS score, median (IQR)	5.0 (3.0–6.0)	6.0 (3.0–8.0)	5.0 (3.0–6.0)	0.111
BATMAN score, median (IQR)	5.0 (3.0–6.0)	6.0 (4.0–7.0)	4.0 (3.0, 6.0)	0.014
GCS score, median (IQR)	8.0 (6.0–9.0)	8.0 (7.0–9.0)	8.0 (6.0–9.0)	0.536
Medical history, n (%)				
Hypertension	90 (75.0)	22 (78.6)	68 (73.9)	0.803
Hyperlipidemia	40 (33.3)	9 (32.1)	31 (33.7)	1
Diabetes mellitus	24 (20.0)	5 (17.9)	19 (20.7)	0.957
Atrial fibrillation	18 (15.0)	4 (14.3)	14 (15.2)	1
Prestroke mRS score at baseline, n (%)				0.075
0	109 (90.8)	23 (82.2)	86 (93.5)	
1	7 (5.8)	2 (7.1)	5 (5.4)	
2	4 (3.4)	3 (10.7)	1 (1.1)	
TIA	1 (0.8)	0 (0.0)	1 (1.1)	1
Coronary heart disease	14 (11.7)	3 (10.7)	11 (12.0)	1
Valvular heart disease	3 (2.5)	0 (0.0)	3 (3.3)	0.782
Biochemical variables, median (IQR)				
Neutrophils, median (IQR)	8.87 (6.96–12.20)	8.35 (7.43–9.97)	9.46 (6.94–12.37)	0.506
Triglycerides, mmol/L, median (IQR)	1.17 (0.78–1.90)	1.32 (0.84–1.96)	1.15 (0.72–1.84)	0.424
Total cholesterol, mmol/L, median (IQR)	4.64 (3.96–5.54)	4.63 (4.00–4.84)	4.68 (3.96–5.58)	0.484
Admission glucose, median (IQR)	7.19 (6.12–8.51)	6.95 (5.79–7.82)	7.28 (6.30–8.97)	0.263
Cause of stroke, n (%)				0.496
LAA	96 (80.0)	23 (82.1)	73 (79.3)	
CE	19 (15.8)	3 (10.7)	16 (17.4)	
Other causes	5 (4.2)	2 (7.1)	3 (3.3)	
General anesthesia, n (%)	NA	NA	32 (34.8)	NA
Intravenous thrombolysis, n (%)	28 (23.3)	5 (17.9)	23 (25.0)	0.598
Pneumonia, n (%)	94 (78.3)	22 (78.6)	72 (78.3)	1
Time variables, min, median (IQR)				
OTI time, min	246.0 (116.0–424.0)	198.0 (87.0–534.0)	260.0 (144.0–412.0)	0.385
OTT time, min	283.0 (162.0–447.0)	229.0 (115.0–584.0)	298.0 (188.0–438.0)	0.372
OTP time, min	NA	NA	363.0 (252.0–492.0)	NA
PTR time, min	NA	NA	107.0 (80.0–151.0)	NA
LiS proportion, n (%)				0.931
Incomplete	63 (52.5)	14 (50.0)	49 (53.3)	
Classic	57 (47.5)	14 (50.0)	43 (46.7)	
Occlusion site, n (%)				0.01
Distal BA	24 (20.0)	2 (7.1)	22 (23.9)	
Middle BA	69 (57.5)	23 (82.1)	46 (50.0)	
Proximal BA	27 (22.5)	3 (10.7)	24 (26.1)	
mTICI score 2b/3, n (%)	72 (60.0)	NA	72 (78.3)	NA
Type of endovascular treatment, n (%)				
Stent retriever thrombectomy	NA	NA	65 (70.7)	NA
Aspiration	NA	NA	5 (5.4)	NA
Balloon angioplasty and/or stenting	NA	NA	10 (10.9)	NA
Intra-arterial medication and/or mechanical fragmentation	NA	NA	12 (13.0)	NA
Combination	NA	NA	63 (68.5)	NA

OTP; onset to puncture; BA, basilar artery; BATMAN, Basilar Artery Treatment and Management; CE, cardioembolism; EVT, endovascular treatment; GCS, Glasgow Coma Scale; IQR, interquartile rage; LAA, large artery atherosclerosis; LiS, locked in syndrome; mRS, modified Rankin Scale; mTICI, modified Thrombolysis in Cerebral Infarction; NIHSS, National Institutes of Health Stroke Scale; OTI, onset to imaging; OTP, onset to puncture; OTT, onset to treatment; pc-ASPECTS, posterior circulation Alberta Stroke Program Early CT Score; pc-CS, posterior circulation collateral system score; PTR, puncture to recanalization; SBP, systolic blood pressure; SMT, standard medical treatment; TIA, transient ischemic attack.

### Outcomes of EVT versus SMT in patients with LiS

**Figure 1:**
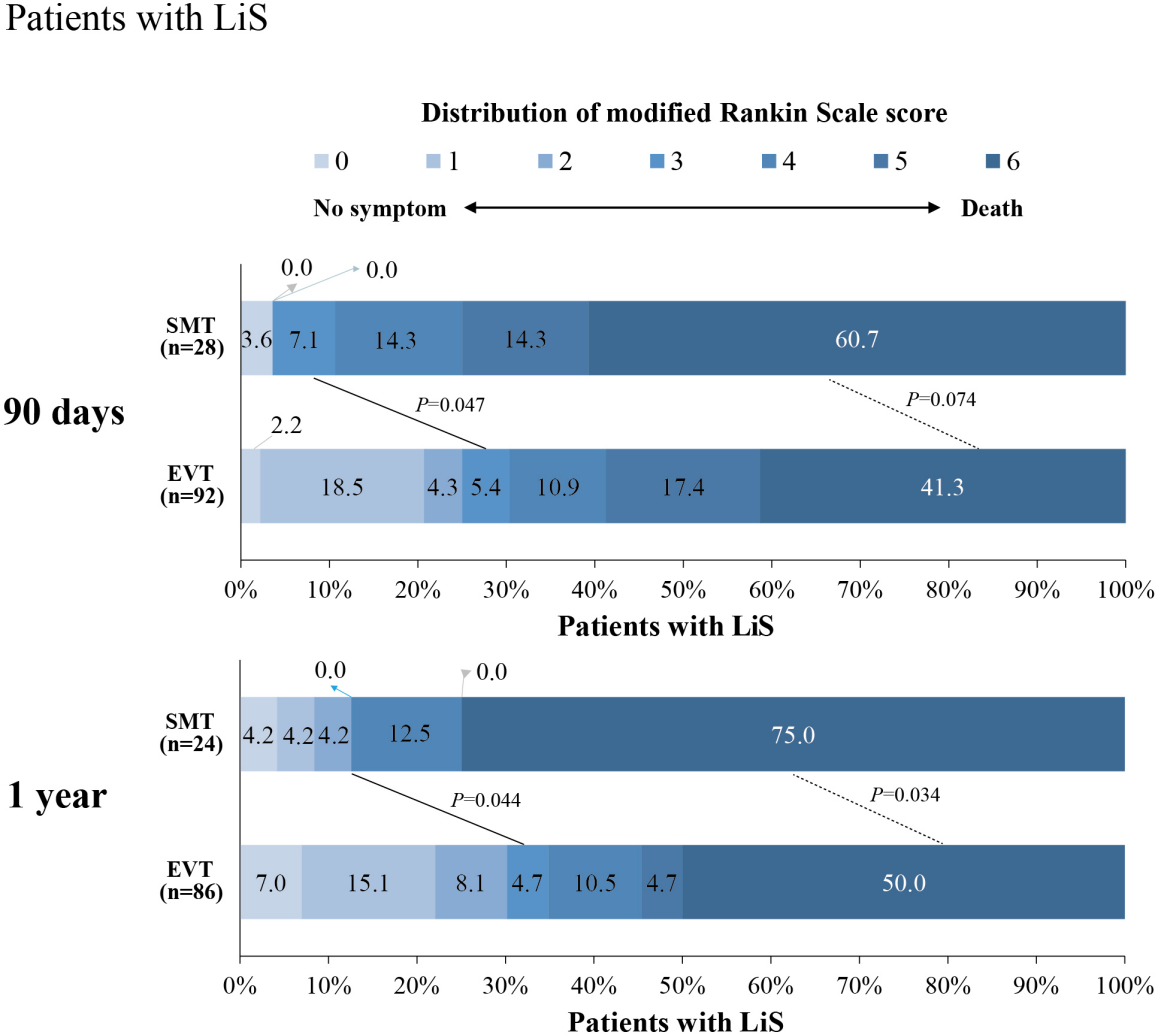
Clinical outcome at 90 days and 1 year. The distribution of modified Rankin scale scores at 90 days and 1 year in patients with LiS is shown. Clinical outcomes at 90 days and 1 year follow-up in patients in the group receiving SMT versus those receiving EVT. EVT, endovascular treatment; LiS, locked-in syndrome; SMT, standard medical treatment.

Analysis of the primary outcome showed that EVT was associated with an improvement in the mRS score (common OR (cOR)=2.68 (95% CI 1.16 to 6.20); p=0.02). The median 90-day mRS score was 5 (IQR 3–6) in the EVT group and 6 (IQR 5–6) in the SMT group. Patients in the EVT group were more likely to have a good outcome (mRS 0–2) (25.0% vs 3.6%; aOR=11.01 (95% CI 1.34 to 90.49); p=0.03) and a favorable outcome (mRS 0–3) (30.4% vs 10.7%; aOR=3.85 (95% CI 1.05 to 14.10), p = 0.04) than those in the SMT group at 90 days (table 2 and figure 1). EVT in patients with acute LiS resulted in functional recovery at 1 year, measured using the mRS score, which was similar to the originally reported results at 90 days. There were no significant differences in survival rates between patients with incomplete and classic LiS (p=0.75) during the follow-up of 1 year (online supplemental figure II).

**Table 2:** Clinical outcomes at 90 days

Characteristics	All patients	SMT	EVT	Unadjusted OR (95% CI)	P value	Adjusted OR* (95% CI)	P value
**Outcomes at 90 days, n (%**)							
mRS score at 90 days, (median (IQR))	5.0 (3.0–6.0)	6.0 (5.0–6.0)	5.0 (3.0–6.0)	2.37 (1.05 to 5.37)†	0.038	2.68 (1.16 to 6.20)†	0.022
mRS score 0–3 at 90 days	31 (25.8)	3 (10.7)	28 (30.4)	3.65 (1.02 to 13.08)	0.047	3.85 (1.05 to 14.10)	0.042
mRS score 0–2 at 90 days	24 (20.0)	1 (3.6)	23 (25.0)	9.00 (1.16 to 69.98)	0.036	11.01 (1.34 to 90.49)	0.026
mRS score 0–1 at 90 days	20 (16.7)	1 (3.6)	19 (20.7)	7.03 (0.89 to 55.07)	0.063	7.91 (0.96 to 64.97)	0.054
Mortality at 90 days	55 (45.8)	17 (60.7)	38 (41.3)	0.46 (0.19 to 1.08)	0.074	0.35 (0.13 to 0.90)	0.03
sICH at 48 hours	7 (5.9)	0 (0.0)	7 (7.7)	NA	0.998	NA	0.998

*The multiple logistic regression test was used to analyze ORs. Outcomes at 90 days used adjusted variables: age, NIHSS baseline, diabetes mellitus.

†Common OR; the primary analysis involved 92 patients in the EVT group and 28 patients in the SMT group. Scores on the mRS of functional disability range from 0 (no symptoms) to 6 (death). The common OR was estimated from an ordinal logistic regression model and indicates the odds of improvement of 1 point on the mRS, with a common OR >1 favoring the endovascular treatment.

EVT, endovascular treatment; mRS, modified Rankin Scale; NIHSS, National Institutes of Health Stroke Scale; OR, odds ratio; sICH, symptomatic intracranial hemorrhage; SMT, standard medical treatment.

Regarding safety outcomes, the rate of symptomatic intracerebral hemorrhage at 48 hours was 7.7% in the EVT group and 0% in the SMT group. EVT was associated with decreased rates of mortality (38 (41.3%) vs 17 (60.7%); aOR=0.35 (95% CI 0.13 to 0.90); p=0.03) at 90 days (table 2). The characteristics and outcomes of LiS subgroups' (incomplete, classic) data are shown in online supplemental table I).

### Predictors of 90-day outcomes in patients with LiS with EVT

Restricting the analysis to the EVT group, patients were dichotomized by functional outcome (ie, favorable vs poor) to investigate the factors associated with outcome after EVT using logistic regression analyses. In the analysis adjusted for prognostic factors, higher baseline pc-ASPECTS (aOR=2.04 (95% CI 1.34 to 3.10); p<0.001), and absence of pneumonia (aOR=0.26 (95% CI 0.08 to 0.90); p=0.03) were significantly associated with increased odds of favorable functional outcome at 90 days. Only lower baseline pc-ASPECTS (aOR=0.52 (95% CI 0.36 to 0.76); p<0.001) was associated with increased odds of 90-day mortality (table 3).

**Table 3:** Predictors of 90-day in patients with LiS with EVT

Variables	mRS score 0–3		Mortality	
	Adjusted OR (95% CI)	P value	Adjusted OR (95% CI)	P value
Age	0.97 (0.93 to 1.02	0.236	1.04 (0.99 to 1.09)	0.073
Diabetes mellitus	0.39 (0.09 to 1.71)	0.212	1.26 (0.39 to 4.02)	0.701
pc-ASPECTS baseline	2.04 (1.34 to 3.10)	<0.001	0.52 (0.36 to 0.76)	<0.001
Pneumonia	0.26 (0.08 to 0.90)	0.033	1.16 (0.35 to 3.82)	0.812

EVT, endovascular treatment; LiS, locked-in syndrome; mRS, modified Rankin Scale; OR, odds ratio; pc-ASPECTS, posterior circulation Alberta Stroke Program Early CT Score.

## Discussion

This multicenter cohort study investigated real-world clinical experiences to evaluate the outcomes associated with EVT in patients with LiS secondary to ABAO. The major finding of this study was the existence of a strong association between EVT and favorable outcomes in patients with LiS secondary to ABAO, with a more than threefold increase in the likelihood of achieving favorable functional outcomes at 90 days, and a significant association between EVT and a lower rate of mortality at 90 days. Moreover, the beneficial effect of EVT in patients with LiS secondary to ABAO was sustained for at least 1 year. In our study, the median mRS score was 5 in the intervention group and 6 in the control group. Although we found that EVT was associated with favorable functional outcomes and decreased mortality among patients with LiS, the finding still reflects the severity of LiS.

LiS is one of the most feared consequences of vertebrobasilar disease, occurring in 14.4% of all patients with BAO in the present study, which is consistent with the findings of a previous report of 10–15%.^14^ The prognosis of patients diagnosed with LiS varies. Earlier literature, primarily relying on autopsy findings, reported that long-term survival without neurological recovery was rare. In 1986, mortality was estimated at 60%, being greatest in the first 4 months and higher in patients with vascular insult than in those with non-vascular causes.^15^ However, several recent case reports have shown that the clinical symptoms of patients have significantly improved.^16–18^ The potential explanation for this difference includes improved therapy, intra-arterial thrombolysis, endovascular mechanical thrombectomy, balloon angioplasty, stenting, or a combination of these approaches, which have been recently administered to these patients. In light of the most disabling and feared stroke syndromes, these initial results are encouraging and indicate that EVT may be a useful emergency treatment for patients with LiS secondary to ABAO. Our results are in line with recent case reports that demonstrated favorable outcomes in patients with LiS secondary to ABAO who underwent EVT.^19^ In addition, similar differences in mortality rate and the percentage of functional outcomes were observed between the groups with incomplete and classic LiS. The mortality rate with EVT was lower in the group with incomplete LiS than in the group with classic LiS, and the percentage of patients with EVT who had favorable functional outcomes was higher in the group with incomplete LiS than in those with classic LiS; however, the differences were not statistically significant.

Clinical predictors associated with a favorable outcome are young age, absence of hypertension, and a history of vertebrobasilar insufficiency.^20^ In this study, a higher baseline pc-ASPECTS, and absence of pneumonia were significantly associated with favorable functional outcomes at 90 days in the EVT group.

Our study showed that in patients with LiS secondary to ABAO, EVT showed clinical benefit compared with SMT alone, and this benefit was evident as early as 90 days after the procedure and continued for up to 1 year. The achievement of favorable functional outcomes at 1 year was significantly higher (by 22.4%) in patients who underwent EVT than in those who underwent SMT. Also, the percentages of patients who achieved excellent, good and favorable outcomes at 1 year were as similar to the 90 days results in both groups. These results may demonstrate the association of intensive nursing care and early rehabilitation programs with favorable outcomes recovery.

This study had several limitations with our results requiring interpretation within the context of the study design. First, this was an observational study, and the associations of EVT and SMT with clinical outcomes were determined in a non-randomized fashion. Therefore, selection bias seemed inevitable in the patient treatment. Moreover, multivariable analyses cannot completely adjust for systematic differences between treatment groups, which is the aim of randomization in clinical trials. Second, the high number of patients who received EVT compared with SMT alone may suggest lack of equipoise among participating centers with regard to the efficacy of EVT in patients with LiS secondary to ABAO. However, our study provides a good representation of daily clinical practice in patients with LiS secondary to ABAO. Despite its limitations, the study still provides one of the best available data regarding EVT for LiS secondary to ABAO.

## Conclusions

Our study provides evidence supporting the safety and efficacy of EVT for patients with LiS secondary to ABAO who can be treated within 24 hours of the estimated occlusion time. Moreover, the beneficial effect of EVT in patients with LiS secondary to ABAO can be sustained for at least 1 year. Baseline pc-ASPECTS and pneumonia were independent factors for clinical outcomes in patients with LiS.

10.1136/jnis-2022-019112.supp2 Supplementary data

## Data Availability

Data are available upon reasonable request.
