# Supplementary material for: Locked-in syndrome responding to endovascular treatment
Source: J Neurointerv Surg. 2022 Aug 19;15(8):808–13. doi: 10.1136/jnis-2022-019112 (PMC10359522; doi:10.1136/jnis-2022-019112)
Supplement: Supplementary data [file jnis-2022-019112supp001.pdf]

Supplementary materials

Supplemental Figure I. Flowchart of patient selection.

Supplemental Figure II. Kaplan-Meier curves Estimates of the Probability of Death during the 1-Year Follow-up.

Supplemental Table I. the baseline characteristics and outcomes of LiS subgroups (incomplete, classical).

Supplemental Figure I. Flowchart of patient selection.

This figure shows the enrollment information of patients in the present study.

Abbreviations: ABAO, acute basilar artery occlusion; LiS, Locked-in syndrome; EVT, endovascular therapy; SMT, standard medical treatment.

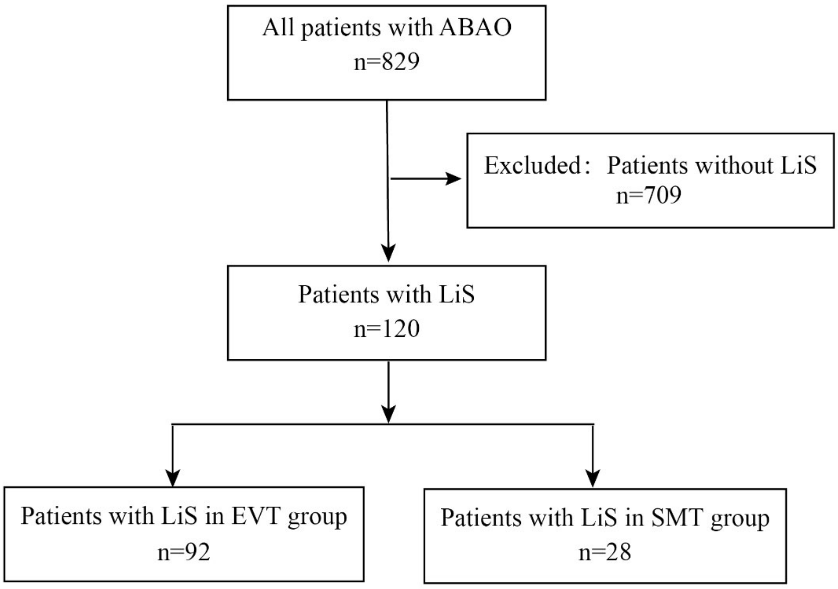

Supplemental Figure II. Kaplan-Meier curves Estimates of the Probability of Death during the 1-Year Follow-up.

Patients in SMT group versus EVT group (A), Patients in Incomplete LiS versus Classical LiS (B).

Abbreviations: LiS, Locked-in syndrome; EVT, endovascular treatment, SMT, standard medical treatment.

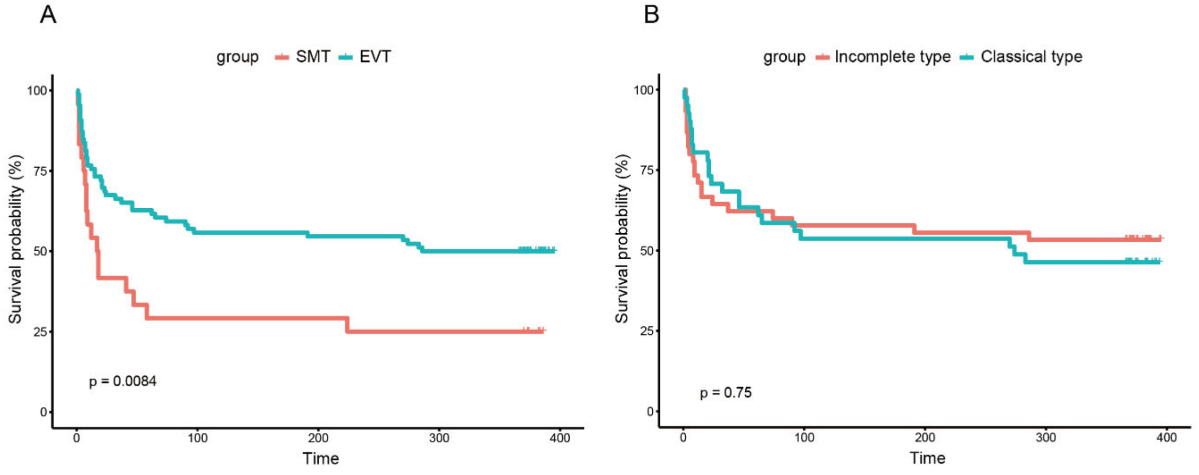

Supplemental Table I. the baseline characteristics and outcomes of LiS subgroups (incomplete, classical).

Variables	SMT group		P value	Variables	EVT group		P value
	Incomplete(n=14)	Classical(n=14)			Incomplete(n=49)	Classical(n=43)	
age (mean (SD)),y	67.00 (13.00)	66.00 (10.00)	0.852	Age, y, median (IQR)	65.00 (55.00-74.00)	66.00 (59.00-75.00)	0.462
Sex, male, n (%)	11 (78.6)	9 (64.3)	0.676	Sex, male, n (%)	36 (73.5)	35 (81.4)	0.513
SBP (mean (SD))	168.00 (32.00)	156.00 (19.00)	0.23	SBP (mean (SD))	155.00 (25.00)	152.00 (24.00)	0.595
Smoking (%)	5 (35.7)	2 (14.3)	0.383	Smoking (%)	18 (36.7)	14 (32.6)	0.841
NIHSS baseline (mean (SD))	17.00 (3.00)	26.00 (2.00)	<0.001	NIHSS baseline (median (IQR))	19.00 (16.00-22.00)	28.00 (25.00-29.00)	<0.001
pc-ASPECTS baseline (median (IQR))	7.00 (6.00- 8.00)	8.00 (7.00- 9.00)	0.112	pc-ASPECTS baseline (median (IQR))	8.00 (7.00- 9.00)	8.00 (7.00- 8.00)	0.076
PC-CS Score (median (IQR))	4.00 (2.00- 6.00)	7.00 (5.00- 8.00)	0.169	PC-CS Score (median (IQR))	5.00 (3.00- 6.00)	4.00 (3.00- 6.00)	0.141
BATMAN Score (median (IQR))	5.00 (3.00- 6.00)	6.00(5.00- 7.00)	0.147	BATMAN Score (median (IQR))	5.00 (3.00- 6.00)	4.00 (3.00- 6.00)	0.214
GCS score, median (IQR)	8.00 (7.00- 10.00)	7.00 (7.00, 8.00)	0.014	GCS score, median (IQR)	9.00 (8.00- 10.00)	6.00 (6.00- 7.00)	<0.001
Medical history, n (%)				Medical history, n (%)			
Hypertension	12 (85.7)	10 (71.4)	0.645	Hypertension	37 (75.5)	31 (72.1)	0.893
Hyperlipidemia	3 (21.4)	6 (42.9)	0.418	Hyperlipidemia	17 (34.7)	14 (32.6)	1
Diabetes mellitus	3 (21.4)	2 (14.3)	1	Diabetes mellitus	10 (20.4)	9 (20.9)	1
Atrial fibrillation	2 (14.3)	2 (14.3)	1	Atrial fibrillation	7 (14.3)	7 (16.3)	1
Pre-mRS, baseline, n (%)			0.441	Pre-mRS, baseline, n (%)			0.24
0	3 (75.0)	3 (42.9)		0	5 (71.4)	4 (50.0)	

1	0 (0)	2 (28.6)	
2	1 (25.0)	2 (28.6)	
Coronary heart disease	2 (14.3)	1 (7.1)	1
Biochemical variables, (median (IQR))			
Triglyceride (median (IQR)), mmol/l	1.22 (0.88- 1.87)	1.38 (0.87- 1.91)	1
Total cholesterol (median (IQR)), mmol/l	4.63 (4.05- 4.80)	4.58 (4.03- 5.72)	0.624
Admission glucose (median (IQR))	7.00 (5.84- 7.98)	6.70 (5.81- 7.54)	0.977
Cause of stroke, n (%)			0.828
LAA	12 (85.7)	11 (78.6)	
CE	1 (7.1)	2 (14.3)	
Other causes	1 (7.1)	1 (7.1)	
General anesthesia, n (%)	NA	NA	NA
Intravenous thrombolysis, n (%)	2 (14.3)	3 (21.4)	1
pneumonia, n (%)	12 (85.7)	10 (71.4)	0.645
Time variables, median, (IQR), min			
OTI time	184.00(90.75 331.00)	217.00 (70.50- 566.00)	0.713
OTT time	212.50 (118.00- 365.50)	253.00 (132.75- 607.25)	0.581

1	1 (14.3)	4 (50.0)	
2	1 (14.3)	0 (0.0)	
Coronary heart disease	3 (6.1)	8 (18.6)	0.129
Biochemical variables, (median (IQR))			
Triglyceride (median (IQR)), mmol/l	1.04 (0.81- 1.81)	1.21 (0.71- 2.06)	0.793
Total cholesterol (median (IQR)), mmol/l	4.68 (4.16- 5.57)	4.58 (3.49- 5.99)	0.608
Admission glucose (median (IQR))	7.18 (6.16- 8.33)	7.32 (6.49- 9.25)	0.352
Cause of stroke, n (%)			0.765
LAA	39 (79.6)	34 (79.1)	
CE	7 (14.3)	7 (16.3)	
Other causes	3 (6.1)	2 (4.7)	
General anesthesia, n (%)	30 (61.2)	29 (69.0)	0.436
Intravenous thrombolysis, n (%)	13 (26.5)	10 (23.3)	0.904
pneumonia, n (%)	34 (69.4)	38 (88.4)	0.028
Time variables, median, (IQR), min			
OTI time	228.00(102.00- 390.000	309.00(207.00- 424.00)	0.054
OTT time	265.00(147.00- 435.00)	350.00(245.00- 447.00)	0.071

OTP time	NA	NA	NA	OTP time	329.00 (262.00-491.00)	385.00 (244.00-495.00)	0.614
PTR time	NA	NA	NA	PTR time	111.00 (85.00-162.00)	98.00 (76.00-141.00)	0.296
Occlusion site, n (%)			0.828	Occlusion site, n (%)			0.334
Distal BA	1 (7.1)	1 (7.1)		Distal BA	9 (18.4)	13 (30.2)	
Middle BA	11 (78.6)	12 (85.7)		Middle BA	25 (51.0)	21 (48.8)	
Proximal BA	2 (14.3)	1 (7.1)		Proximal BA	15 (30.6)	9 (20.9)	
mTICI score 2b/3, n (%)	NA	NA	NA	mTICI score 2b/3, n (%)	37 (75.5)	35 (81.4)	0.668
**Clinical outcomes, n (%)**				**Clinical outcomes, n (%)**			
mRS 0-3 at 90 days	1 (7.1)	2 (14.3)	1	mRS 0-3 at 90 days	17 (34.7)	11 (25.6)	0.471
mRS 0-2 at 90 days	1 (7.1)	0 (0.0)	1	mRS 0-2 at 90 days	16 (32.7)	7 (16.3)	0.117
mRS 0-1 at 90 days	1 (7.1)	0 (0.0)	1	mRS 0-1 at 90 days	13 (26.5)	6 (14.0)	0.219
Mortality at 90 days	8 (57.1)	9 (64.3)	1	Mortality at 90 days	19 (38.8)	19 (44.2)	0.754
sICH	0	0	NA	sICH	2 (4.2)	5 (11.6)	0.347

Abbreviation: BA, basilar artery; CE, cardioembolism; EVT, endovascular treatment; IQR, interquartile rage; LAA, large artery atherosclerosis; LiS, locked in syndrome; mRS, modified Rankin Scale; mTICI, modified Thrombolysis in Cerebral Infarction; NIHSS, National Institutes of Health Stroke Scale; OTI, onset to imaging; OTP; onset to puncture; OTT, onset to treatment; pc-ASPECTS, posterior circulation Alberta Stroke Program Early Computed Tomography Score; PC-CS score, posterior circulation collateral system score; PTR, puncture to recanalization; SBP, systolic blood pressure; sICH, Symptomatic intracranial hemorrhage; SMT, standard medical treatment; TIA, Transient Ischemic Attack.
